# Bio-Active Compounds from *Teucrium* Plants Used in the Traditional Medicine of Kurdistan Region, Iraq

**DOI:** 10.3390/molecules27103116

**Published:** 2022-05-12

**Authors:** Fuad O. Abdullah, Faiq H. S. Hussain, Abdullah Sh. Sardar, Gianluca Gilardoni, Zaw Min Thu, Giovanni Vidari

**Affiliations:** 1 Department of Chemistry, College of Science, Salahaddin University-Erbil, Erbil 44001, Kurdistan Region, Iraq; 2 Department of Pharmacognosy, Faculty of Pharmacy, Tishk International University, Erbil 44001, Kurdistan Region, Iraq; 3 Department of Medical Analysis, Faculty of Applied Science, Tishk International University, Erbil 44001, Kurdistan Region, Iraq; faiq.hussain@tiu.edu.iq; 4 Department of Biology, College of Education, Salahaddin University-Erbil, Erbil 44001, Kurdistan Region, Iraq; abdullah.sardar@su.edu.krd; 5 Departamento de Química, Universidad Técnica Particular de Loja, Loja 110107, Ecuador; ggilardoni@utpl.edu.ec; 6 Department of Chemistry, Kalay University, Kalay 03044, Myanmar; zawminthu@kalayuniversity.edu.mm; 7 Dipartimento di Chimica, Università di Pavia, 27100 Pavia, Italy

**Keywords:** *Teucrium*, Kurdish traditional medicine, bio-active secondary metabolites, biological activities, hepatotoxicity

## Abstract

Herbal medicine is still widely practiced in the Kurdistan Region, Iraq, especially by people living in villages in mountainous regions. Seven taxa belonging to the genus *Teucrium* (family Lamiaceae) are commonly employed in the Kurdish traditional medicine, especially to treat jaundice, stomachache and abdominal problems. We report, in this paper, a comprehensive account about the chemical structures and bioactivities of most representative specialized metabolites isolated from these plants. These findings indicate that *Teucrium* plants used in the folk medicine of Iraqi Kurdistan are natural sources of specialized metabolites that are potentially beneficial to human health.

## 1. Introduction

**Figure 1:**
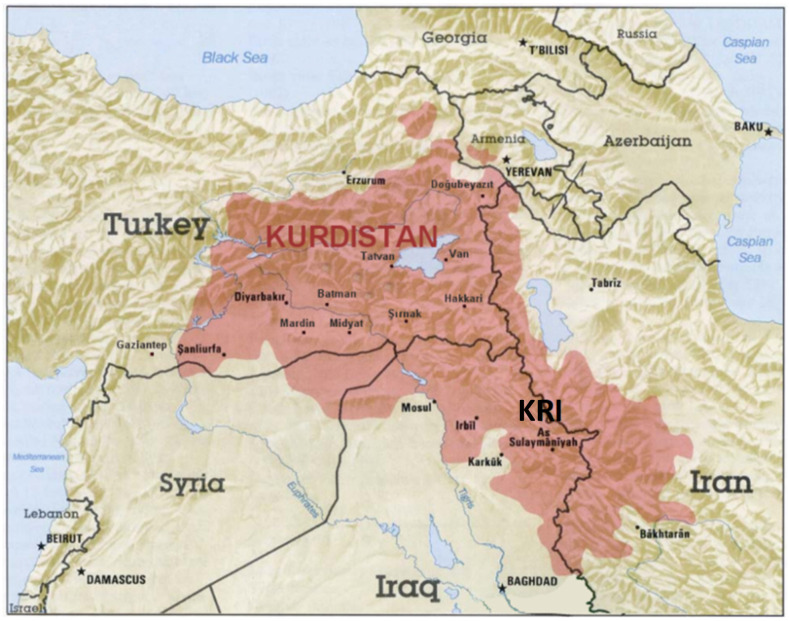
The approximate map of the Kurdish-populated region (“Kurdistan”) which includes parts of Turkey, Syria, Iraq and Iran (taken and adapted from https://www.bing.com/images/search for Kurdistan, latest accessed on 24 April 2022).

Nature is a major source of current medicines, and many (semi)synthetic drugs have been developed from the study of bioactive compounds isolated from extracts of plants used in traditional medicines of different countries [1]. Kurds—the peoples living in Turkey, Iran and other East Asian countries—have been practicing traditional medicine from a time immemorial. In fact, the practices of medicinal plant uses are transmitted orally as a part of the Kurdish cultural heritage. Moreover, the popularity of herbal remedies has increased among Kurds during the last two decades, in part because of the high cost of synthetic drugs, which are mainly imported from abroad. Thus, in rural communities, herbal remedies are the first choice for the treatment of many diseases and practitioners of traditional medicine dispense primary care. However, also in the markets of the big cities, such as in the Qaysary Market located in the centre of Erbil (Irbil), the capital of the Kurdistan Region, Iraq (KRI) (Figure 1), several shops sell different natural medicinal products [2]. It is interesting to note that about 64% of these products have their origin outside Iraqi Kurdistan, being imported from countries as far away as India, Spain and Libya, while only 36% come from different districts within the Kurdistan Region [2].

Despite the wide use of herbal remedies, a limited number of papers have been published in Kurdistan concerning the structures and bioactivities of specialized metabolites isolated from native plants [3,4,5,6,7,8,9,10,11,12,13,14,15,16,17,18,19]. With the aim to add more value to the medicinal plants growing in the Kurdistan Region, Iraq, to sustain their uses with scientific evidence of the efficacy and to foster further investigations, we report in this paper a comprehensive account of the structures and bioactivities of most representative compounds isolated from *Teucrium* taxa used in the traditional medicine of the Kurdistan Region.

Iraqi Kurdistan or Southern Kurdistan refers to the Kurdish-populated part of northern Iraq (Figure 1). It is considered one of the four parts of “Kurdistan” in Western Asia, which also includes parts of southeastern Turkey, northern Syria and northwestern Iran (Figure 1). Much of the geographical and cultural region of Iraqi Kurdistan is part of the Kurdistan Region, Iraq (KRI), an autonomous region ruled by the Kurdish Regional Government, which is recognized by the Constitution of Iraq. As with the rest of Kurdistan, and unlike most of the rest of Iraq, the region is inland and mountainous.

## 2. Ethnobotanical Data about the *Teucrium* Species Growing in the Kurdistan Region, Iraq

*Teucrium* L. is the second-largest genus of the subfamily Ajugoideae in the family Lamiaceae (Labiatae), with a subcosmopolitan distribution and more than 430 taxa with accepted names [20,21]. Mild climate regions, such as the Mediterranean and the Middle East areas, contain about 90% of the total *Teucrium* taxa [22]. Most *Teucrium* have been used in several traditional medicines for thousands of years, and have different potential applications, from pharmaceutical to food industries, primarily due to the high content of specialized metabolites with significant biological activities. It is interesting to note that the name *Teucrium* derives from the Greek terms “τευχριον—teúcrion”, in honor of an ancient Trojan king who, according to the Roman historiographer Pliny, was the first one to utilize these plants for medical purposes. In fact, various compounds isolated from *Teucrium* taxa have shown antipyretic, diuretic, diaphoretic, genotoxic, antioxidant, antibacterial, antifungal, antiviral, anticancer, cholesterol-lowering, hypoglycemic, anti-malaria, spasmolytic, anti-inflammatory and even antifeedant properties [23,24,25,26,27]. A comprehensive study on the entire *Teucrium* genus, reviewing the publications conducted in the last two decades was published recently, during the final preparation of this paper [28]. 

Seven *Teucrium* taxa are used in the traditional medicine practiced in the Kurdistan Region [22,23], where these plants grow abundantly in certain areas. In Table 1, we report the botanical names, the traditional uses and the growth places in Iraqi Kurdistan. Decoctions and infusions are the most frequently used procedures in the preparation of traditional remedies from these *Teucrium* plants.

In addition, to the plants listed in Table 1, *T. multicaule* Montbret & Aucher ex Benth., *T. procerum* Boiss. & C. I. Blanche, *T. pruinosum* Boiss., *Teucrium orientale* ssp. *taylorii* (Boiss.) Rech.f. (synonym *T. taylorii* Boiss.) grow across the rest of Iraq [29,30].

## 3. Phytochemistry and Ethnopharmacology of *Teucrium* Taxa Used in the Traditional Medicine of the Kurdistan Region—Iraq Methods for the Literature Search

In this inaugural paper on the phytochemical and ethnopharmacological aspects of *Teucrium* taxa used in the traditional medicine of Iraqi Kurdistan (Table 1), the pertinent literature was reviewed from 1970 until the end of November 2021 using the Reaxys, Google Scholar, ScienceDirect, Scifinder, PubChem and PubMed databases. Terms and keywords used for the survey have been the “botanical name of each taxon”, “Germander”, “Kurdistan traditional medicine”, “Kurdistan *Teucrium*” and “*Teucrium* metabolites”. Since the plants listed in Table 1 are also used in the traditional medicines of other countries, especially in the Middle East, for the sake of completeness, we extended our survey to the entire literature on the phytochemistry and ethnopharmacology of most representative specialized metabolites isolated from the plants listed in Table 1. In fact, most scientific data about these plants were gathered outside Kurdistan.

The molecular structures of identified representative compounds, divided in accordance with the biogenesis, are shown in Figure 2, Figure 3, Figure 4, Figure 5, Figure 6, Figure 7, Figure 8, Figure 9, Figure 10, Figure 11 and Figure 12, whereas, in addition to Table 1, traditional uses and data of biological activities in vitro are reported in Table 2.

### 3.1. Phytochemical Aspects

A survey of the literature reveals that more than 290 compounds have been identified in the essential oils and non-volatile extracts from the *Teucrium* taxa used in the folk medicine of the Kurdistan Region, Iraq (Table 1). They include mono-, sesqui-, di- and triterpenoids, steroids, flavonoids, phenylethanoid glycosides, etc. 

No phytochemical investigation has yet been dedicated to *T. rigidum* Benth. Essential oils (EOs) are the most analyzed phytochemical parts of the other taxa listed in Table 1, except the oil from *T. oliverianum*. EOs were isolated by conventional hydrodistillation in a Clevenger apparatus and were analyzed by standard GC-FID and GC-MS techniques. Both polar (Carbowax 20M) and non-polar (OV1 and SE 30) columns were used. EOs can, in general, be divided between those where the main components are sesquiterpene hydrocarbons, such as β-caryophyllene (**166**) and germacrene D (**167**), as the oil from *T. parviflorum*, and the oils where monoterpene hydrocarbons, such as α- (**174**) and β-pinene (**175**) predominate, as the oil from *T. melissoides*. There are significant qualitative and quantitative differences between the EOs of the different taxa and this variability is even intraspecific, which is probably due to the genetic, differing chemotypes, drying conditions, mode of oil distillation, extraction and/or storage, and geographic or climatic factors. The compositions of the EOs isolated from specimens of *T. chamaedrys* and *T. polium* collected in different countries, are typical examples of such variability [26]. The high content of menthofuran (**188**) in the EO from *T. scordium* subsp. s*cordioides* collected in Serbia [132] indicates that this terpenoid can be considered a chemotaxonomic marker of the oil.

Regarding the structures of non-volatile secondary metabolites, those occurring in *T. melissoides* and *T. parviflorum* are still unknown and only a few studies have been dedicated to the contents in extracts from *T. oliverianum* and *T. scordium* subspecies *scordioides*. Instead, the phytochemical aspects of *T. chamaedrys* and *T. polium* have been subjected to several investigations. These studies have possibly been promoted by the worldwide occurrence and widespread medicinal uses of the two plants.

Among monoterpenoids, the presence of iridoids, as aglycones and glycosides, in *T. chamaedrys, T. oliverianum* and *T. polium* extracts, confirms the observation that they are chemotaxonomic markers of the Lamiaceae family and have been recognized in several genera of the Ajugoideae and Lamioideae subfamilies.

Non-volatile sesquiterpenoids **142–159** (Figure 10) were so far isolated from only *T. polium* [81,88,95]. Most of them belonged to the eudesmane, cadinane and germacrane families.

Diterpenes include representatives of the *abeo*-abietane and *neo*-clerodane families. Compounds of the first group (**129–141**) have been isolated only from *T. polium* [90]; on the other hand, *T. chamaedrys*, *T. oliverianum* and *T. polium* are rich sources for *neo*-clerodane diterpenoids (Figure 3, Figure 4 and Figure 5). Indeed, these compounds probably represent the most abundant family of specialized metabolites occurring in *Teucrium* taxa and are considered the chemotaxonomic markers of the genus [134]. The reason of the wide distribution and conservation of *neo*-clerodanes might be likely due to their potential allelopathic properties [39], a pronounced protective role against herbivore predators and the general antifeedant activity [25,134]. Thus, the last effects may have economic importance against *Lepidopterous* pests [135]. Most *neo*-clerodanes shown in Figure 3, Figure 4 and Figure 5 contain a characteristic *trans*-fused decalin core with an α-spiro 4-(3-furyl)-γ-butyrolactone-based side chain attached to C-9. Representative structures are compounds **10–26**. However, the lactone ring may be absent, as in **47–53** and even the furan moiety may be missing, as in syspirensin B (**33**). Except for teupolins VII (**75**) and VIII (**76**), the carbon C-6 is oxygenated. Other frequently oxygenated carbons are C-2, C-3, C-7, C-12, C-18, C-19 and, rarely, C-10, as in *neo*-clerodanes **45**, **47**, **50** and **51**. Moreover, the presence of an epoxide, as in **23** and **38**, an oxetane, as in **27**, **34** and **67**, a tetrahydrofuran, as in **47** and **81**, a hemiacetal or an acetal, as in **26**, **58**, **60**, **73**, **79**, a γ-lactone, as in **13**, **16**, **29**, **34**, **45**, or a δ-lactone ring, as in **28**, **83**, **86**, add to complicate the chemical structures of these diterpenoids.

The NMR data of representative *Teucrium* sesquiterpenes, *neo*-clerodane and *abeo*-abietane diterpenoids are thoroughly discussed in reference [25].

Readers must be aware that some ambiguities exist in the literature about the names and even the stereochemistry of a few *neo*-clerodane diterpenoids isolated from *Teucrium*. Typical examples are compound **16**, teucvidin (**18**) and syspirensin A (**32**). These uncertainties depend on the fact that chemical structures were based mainly on the interpretation of NMR spectra, while only a few ones were firmly confirmed by X-ray analysis [56] or stereoselective total synthesis [53]. The chemical structures depicted in Figure 3, Figure 4 and Figure 5 are reported in the most recent publications.

The group of sterols and triterpenoids comprises compounds widely distributed in plants, such as β-sitosterol, campesterol and oleanolic acid, but also novel triterpene saponins, poliusaposides A-C (**163–165**), that were isolated from a MeOH extract of *T. polium* aerial parts.

Flavonoids, in glycosidic and aglycone forms (Figure 6), are relatively abundant in *T. chamaedrys* and *T. polium*, while only a couple of compounds, **87** and **92**, occurred in *T. oliverianum* extracts [72,73]. The most characteristic flavonoids are the flavones apigenin (**90**) and luteolin (**95**) and a small group of derivatives, including 4′- and 7-*O*-glycosides and some 6-methoxy flavones. It is worth noting that luteolin 7-*O*-β-D-(5-*O*-syringyl)apiofuranosyl-(1→2)-β-D-glucopyranoside (**106**), isolated from a MeOH extract of *T. polium* leaves, contains an unprecedented structure [92]. Other classes of flavonoids are rarely represented in the extracts. In fact, the only flavanone isolated so far has been naringenin (**99**), from *T. chamaedrys* subsp. *chamaedrys*, while the only isolated flavonol *O*-glycoside has been rutin (**100),** from extracts of *T. polium* [87,90]. A rare 6,8-di-*C*-glucoside, vicenin-2, was also isolated from *T. polium* [118].

Among other phenolic derivatives, only a couple of lignans (**125** and **126**) have been isolated [96]. This finding is quite interesting because lignans, phenylpropanoid dimers, are produced as a result of plant defense against stress. On the other hand, *T. chamaedrys* and *T. polium* are good sources of phenylethanoid glycosides, the verbascoside derivatives **107–117** (Figure 7).

### 3.2. Bioactivity and Pharmacological Properties

Antioxidant and free radical-scavenging properties have been determined for most extracts and isolated phytochemicals described in this review, including EOs [26,76], iridoid glycosides [85], *abeo*-abietanes [90], phenyl ethanoid glycosides [38,85] and flavonoids [85,88,92]. However, such antioxidant action has usually been evaluated through various standard in vitro assays, in cell-free systems, which included cupric reducing antioxidant capacity (CUPRAC) assay [88], DPPH scavenging (DPPH), reducing power (RP), xanthine oxidase inhibitory effect (XOI) and antioxidant activity in a linoleic acid system (ALP) [85]. Therefore, these evaluations have limited pharmacological relation and limit the validation of the established biological action. Moreover, the antioxidant activity of an extract may be due to a synergistic effect of the various components through different antioxidant mechanisms,

Antibacterial and other bioactivities of EOs have been described in detail in a previous review [26]. An interesting potential application of the antimicrobial power of EOs, for example that from *T. polium*, concerns the implementation as preservative additives in the food industry in order to fight microbial contaminations and development [26,36] and lipid oxidation [123].

A study of the relationship between structure and antioxidant effects has been performed on verbascoside (**114**) and derivatives (Figure 7). The activity varied, depending on the glycosylation and methylation patterns. It was observed that increasing sugar units with accompanying free-phenolic-hydroxyl pairs increased antioxidant activity, while hydroxyl methylation decreases this effect [88]. Thus, poliumosides **110** and **116** showed the highest antioxidant capacity. It was suggested that phenolic hydroxyl pairs form hydrogen bond between adjacent groups that can stabilize phenoxy radical intermediates. Instead, hydroxyl methylation destabilizes the intermediate by disrupting hydrogen bonding [88]. Noteworthy, compounds **114** and **116** with free *ortho*-dihydroxyl groups showed higher activity than the positive controls, trolox and α-tocopherol [88]. A similar trend was observed with the radical scavenging activity of a group of flavonoids isolated from *T. polium* [85,92]. Luteolin (**95**) and luteolin-based compounds with a free *ortho*-dihydroxy group in ring B elicited a massive reduction of the radical species, whereas luteolin 4′-*O*-glucoside (**93**) and apigenin (**90**) showed very low or no radical scavenging and reducing properties. This finding underlined that the structural feature responsible for the observed activity is the presence of a C-2–C-3 double bond, a carbonyl group at C-4 and, more importantly, a free *ortho*-dihydroxy (catechol-type) substitution in the flavone B-ring. The authors suggested that the formation of flavonoid phenoxy radicals may be stabilized by the mesomeric equilibrium to *ortho*-semiquinone structures [85]. In this regard, the higher scavenging and reducing activities of compounds **110** and **116**, with respect to flavonoid glycosides, could be attributed to the presence of a second phenolic ring in the caffeoyl residue with an *ortho*-dihydroxy group [85]. Flavonoid aglycones showed more potent antiradical action than their corresponding *O*-glycosides in the A or B ring and a disaccharide moiety bound to the C-7 of the A-ring weakens the antiradical effect, as observed for luteolin 7-O-rutinoside (**101**) and luteolin 7-*O*-neohesperidoside (**102**), compared to luteolin 7-*O*-glucoside (**103**) and luteolin (**95**). In contrast to these findings, *T. polium* flavonoids **101–103** showed a lower xanthine oxidase inhibitory (XOI) activity than compound **93**. It was suggested that the presence of a free C-4′ hydroxyl group in compounds **101–103** makes them more easily ionizable and, therefore, less able than flavonoid **93** to interact with the hydrophobic channel, which is the main access to XO active site [85].

Among other bioactive metabolites, the iridoid harpagide (**1**) exerted a wide number of biological activities such as cytotoxic, anti-inflammatory, anti-osteoporotic and neuroprotective effects [136]. Similarly, 8-*O*-acetylharpagide (**2**) showed vasoconstrictor [137], antitumoral, antiviral, antibacterial and anti-inflammatory activities. Notably, an inconsistent connection between anti-tumor and antioxidant/radical scavenging activity was observed for the iridoids **2** and teucardoside (**3**) and poliumoside (**116**), which resulted in varied effects on several cancer cell lines. This finding is consistent with some in vivo studies [88]. Moreover, iridoids **2** and **3** exhibited an extraordinary ability to inhibit lipid peroxidation [85]. It was suggested that the presence of a free hydroxyl group on C5 of the iridoid **2** could be responsible for the higher antioxidant ability than compound **3** [85].

The saponin glycosides poliusaposides A-C (**163–165**) were evaluated for anticancer effects by a National Cancer Institute 60 human tumor cell line screen (http://dtp.nci.nih.gov/branches/btb/ivclsp.html, latest accessed on 24 April 2022) [98]. Poliusaposide C (**165**) completely inhibited the growth of a breast (MDA-MB-468) and colon cancer line (HCC-2998) and partially inhibited the growth of a colon (COLO 205), renal (A498) and melanoma cancer (SK-MEL-498) cell line [98]. The other two saponins were considerably less active. By analogy with previously reported bidesmosidic saponins, it was suggested that the increased activity of **165**, compared to **163** and **164**, was linked to the presence of multiple apiose units, the apiose branching in the oligosaccharide moiety attached to C-28, the difference in glycan chain polarity and the increased aglycone hydroxylation, due to the reduction of the triterpenoid carboxylic acid group to a primary alcohol [98].

Verbascoside (**114**) exhibited anti-inflammatory, immunosuppressive, anti-infective and protein kinase C inhibitory properties [138]; moreover, compound **114** and forsythoside B (**111**) exerted strong neuroprotective and antiseptic effects [32,139].

The hypoglycemic effect of *T. polium* has been accounted for by its constituents that increase insulin release [105]. In this context, the effect of the major flavonoids occurring in *T. polium* extract, rutin (**100**) and apigenin (**90**), on insulin secretion at various glucose concentrations was investigated [89]. The two flavonoids demonstrated protective effects on β-cell destruction in a model of streptozotocin-induced diabetes, due to the antioxidant activity [89]. Among other flavonoids isolated from *T. polium*, cirsiliol (**87**) showed good relaxant, sedative and hypnotic effects [32]. Moreover, 3′,4′,5-trihydroxy-6,7-dimethoxyflavone and 5,6,7,3′,4′-pentahydroxyflavone, carrying a 3′,4′-dihydroxy B-ring pattern, showed an interesting inhibitory activity against the biofilm-forming *Staphylococcus aureus* strain AH133. It was suggested that due to the antibacterial activity, these flavonoids can potentially be used for coating medical devices such as catheters [96]. Antibacterial activity of the crude extract of *T. polium*, as well as of isolated flavonoid salvigenin (**94**) and sesquiterpenoids **151** and **154**, was observed with *Staphylococcus aureus* anti-biofilm activity in the low μM range [95]. It should be noted that biofilm formation is a physical strategy that bacteria employ to effectively block the penetration and toxicity of antibiotics. Thus, blocking or retarding the formation of biofilms improves the efficacy of antibiotics.

Shawky in a network pharmacology-based analysis showed that *T. polium* had potential anti-cancer effects against A375 human melanoma cells, TRAIL-resistant Huh7 cells and gastric cancer cells, likely due to luteolin (**95**) occurrence in the plant. In fact, luteolin inhibited the proliferation and induced the apoptosis of A375 human melanoma cells by reducing the expression of MMP-2 and MMP-9 proteinases through the PI3K/AKT pathway [140].

Moreover, it was found that the aqueous extract of *T. polium* aerial parts, given intraperitoneally, reduced significantly the serum levels of cholesterol and triglycerides in hyperlipidemic rats. It was suggested that the presence of flavonoid and terpenoid constituents may play a role in the observed hypolipidemic effects [106].

Although many crude extracts or partially purified fractions from *Teucrium* have showed various beneficial biological and pharmacological effects (see the literature cited in Table 2), the use of herbal remedies prepared from *Teucrium* plants should be considered with caution unless the safety has been demonstrated by rigorous scientific evidence. A paradigmatic example is *T. chamaedrys*, commonly known as ‘germander’, which has long been used as dietary supplement for facilitating weight loss or as a hypoglycemic aid. However, acute and chronic hepatitis and even fatal cirrhosis were observed in patients who had consumed the plant as a tea for 3–8 weeks [35,141]. Subsequently, similar hepatotoxicity was observed with other members of the *Teucrium* genus, including the widely used *T. polium* [142,143]. These toxic effects have mainly been associated with the presence of abundant *neo*-clerodane diterpenoids, especially teucrin A (**19**) [54]. In fact, Lekehal and collaborators, while studying the in vivo hepatotoxicity of teucrin A (**19**) and teuchamaedryn A (**20**), as well as the furano diterpenoid fraction of *T. chamaedrys*, demonstrated that the furan ring of *neo*-clerodane diterpenoids is bioactivated by CYP3A (cytochrome P450 enzymes) into electrophilic metabolites that covalently bind to hepatocellular proteins, deplete GSH and cytoskeleton associated protein thiols and lead to formation of plasma membrane blebs and apoptosis in rat hepatocytes [42,142]. 1,4-Enedials or possible epoxide precursors are the likely reactive toxic metabolites [143]. In contrast to these findings, 18 *neo*-clerodane diterpenes containing a 3-substituted furan ring, isolated from *T. polium*, showed low toxicity at the highest test concentration (200 μM) against HepG2 human cells, which provided a useful model to study the function of the CYP3A4 enzyme [100]. The authors of the study suggested that a high concentration of *neo*-clerodanes in the crude extract or synergistic effect of the *neo*-clerodanes with one another or with other phytochemicals present in the plant might produce hepatotoxic effects. Other lines of evidence implicate immune-mediated pathways in initiating liver injury. In other cases, autoantibodies were present [142,144].

## 4. Conclusions

**Figure 2:**
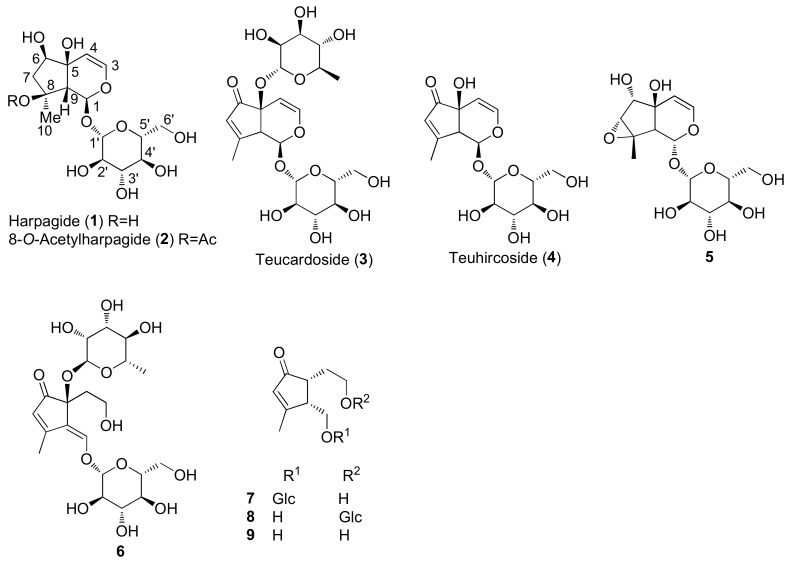
Representative iridoids and iridoid glycosides isolated from *Teucrium chamaedrys, Teucrium oliverianum* and *Teucrium polium*.

**Figure 3:**
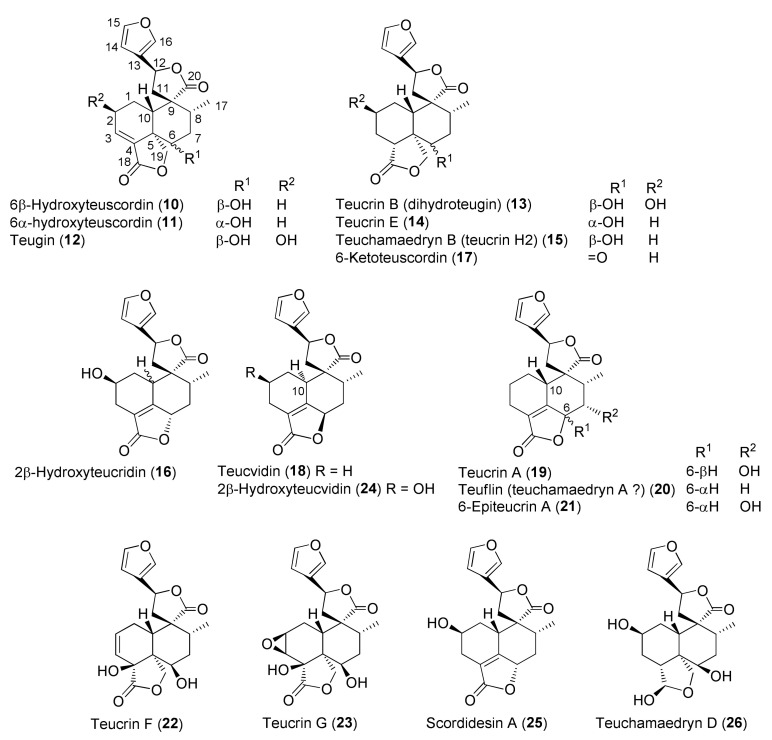
Representative neo-clerodane diterpenoids isolated from *Teucrium chamaedrys* and *Teucrium scordium* subspecies *scordioides*.

**Figure 4:**
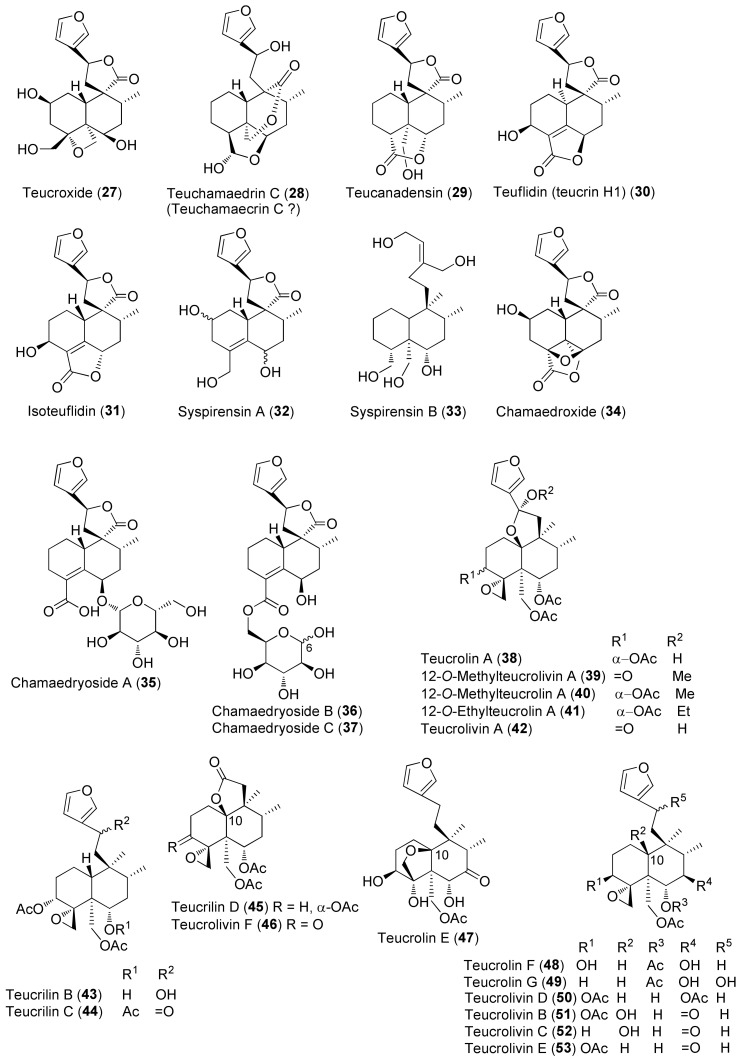
Representative *neo*-clerodane diterpenoids isolated from *Teucrium chamaedrys* and *Teucrium oliverianum*.

**Figure 5:**
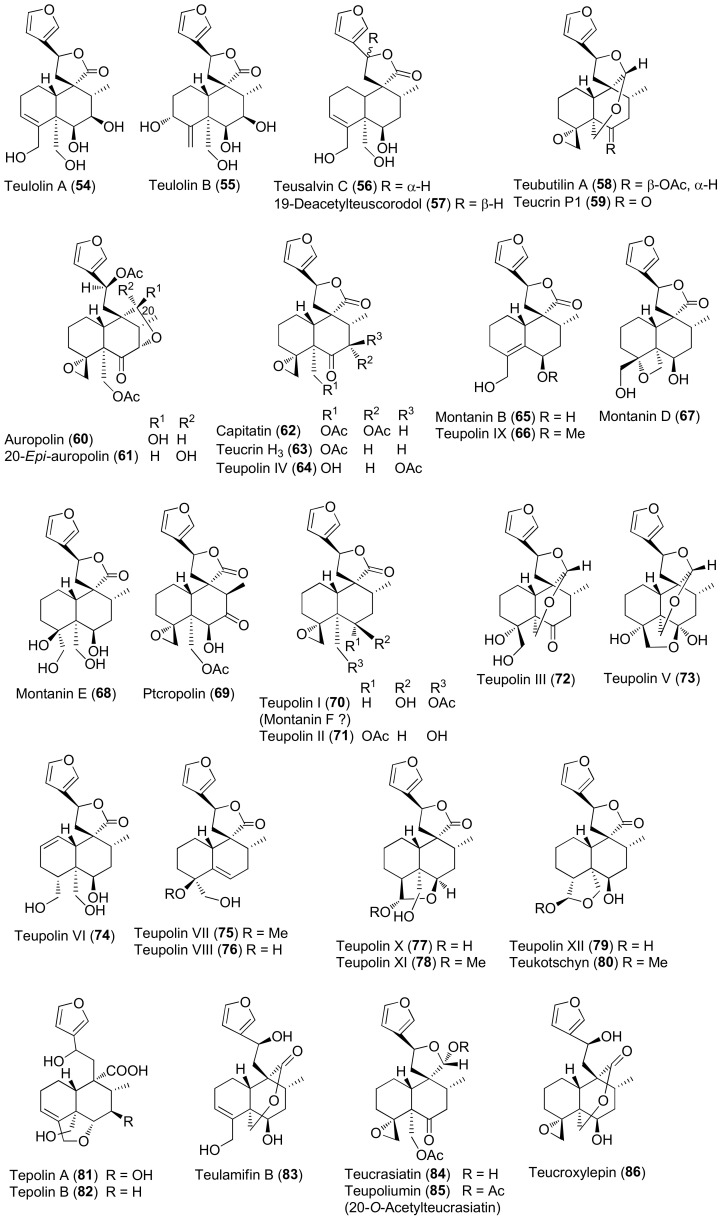
Representative neo-clerodane diterpenoids isolated from *Teucrium polium*.

**Figure 6:**
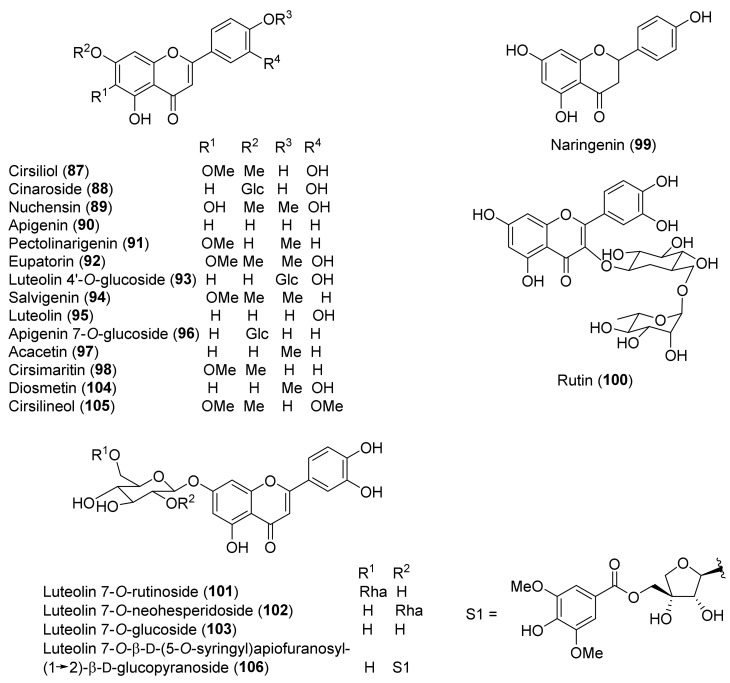
Representative flavonoids isolated from *Teucrium chamaedrys*, *Teucrium oliverianum* and *Teucrium polium*.

**Figure 7:**
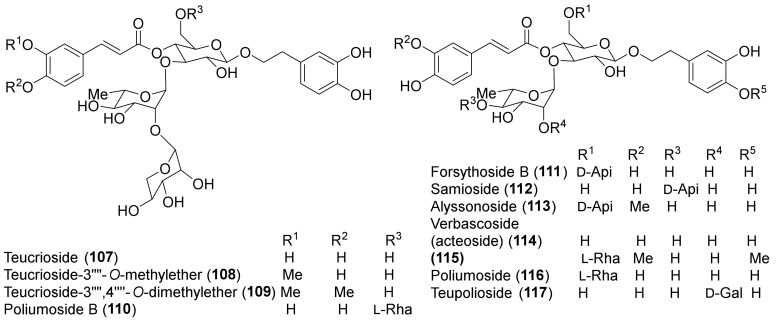
Representative verbascoside derivatives isolated from *Teucrium chamaedrys* and *Teucrium polium*.

**Figure 8:**
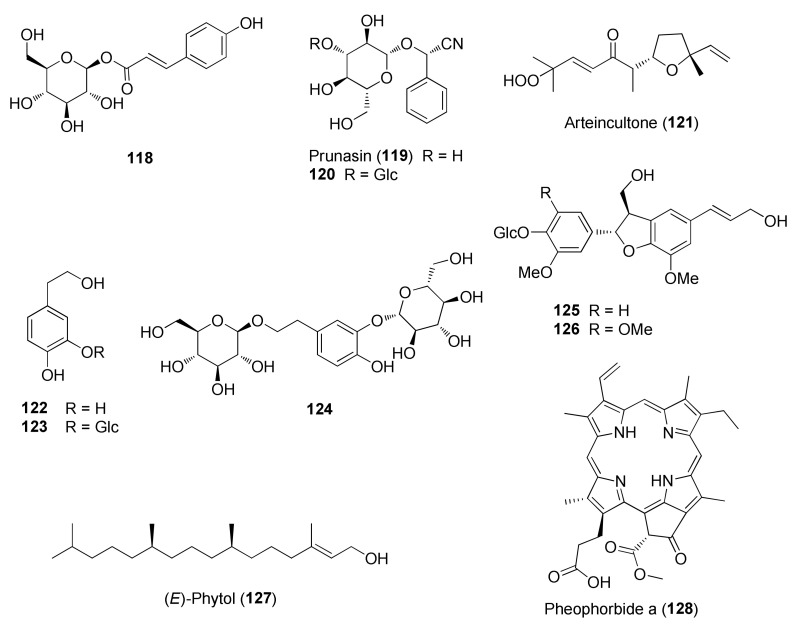
Representative phenolics, lignans and miscellaneous compounds isolated from *Teucrium chamaedrys* and *Teucrium polium*.

**Figure 9:**
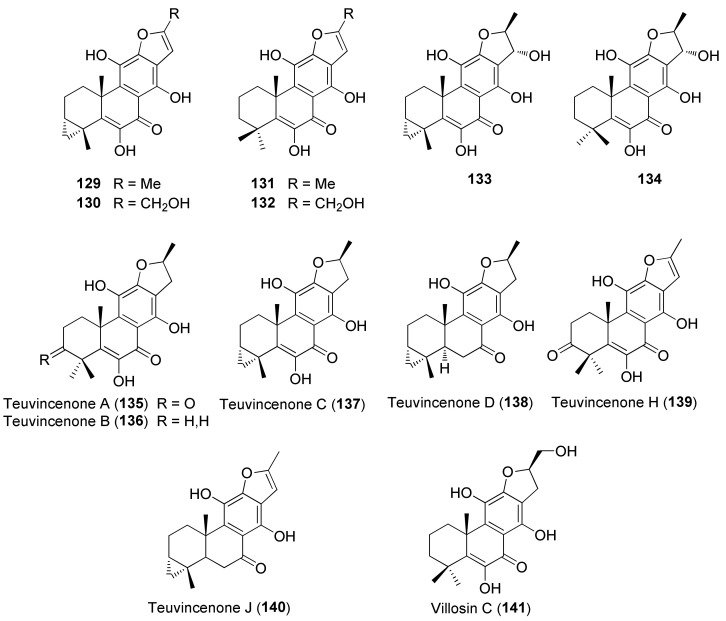
Representative *abeo*-abietane diterpenoids isolated from *Teucrium polium*.

**Figure 10:**
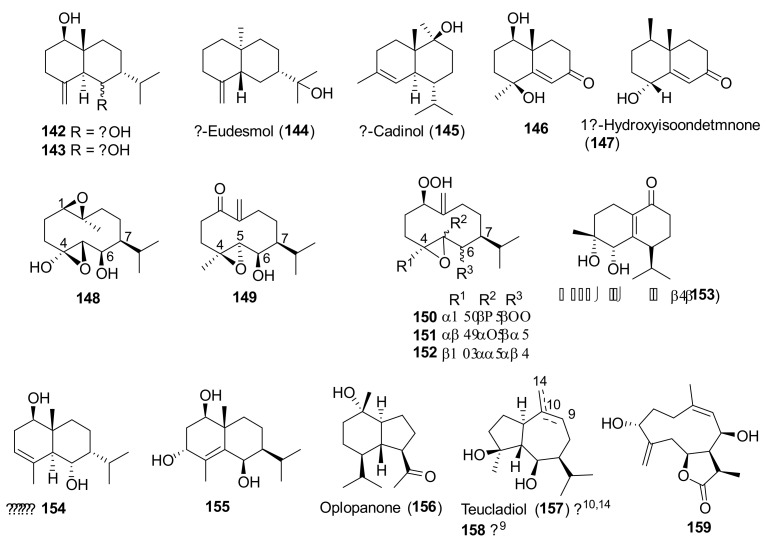
Representative sesquiterpenoids isolated from *Teucrium polium*.

**Figure 11:**
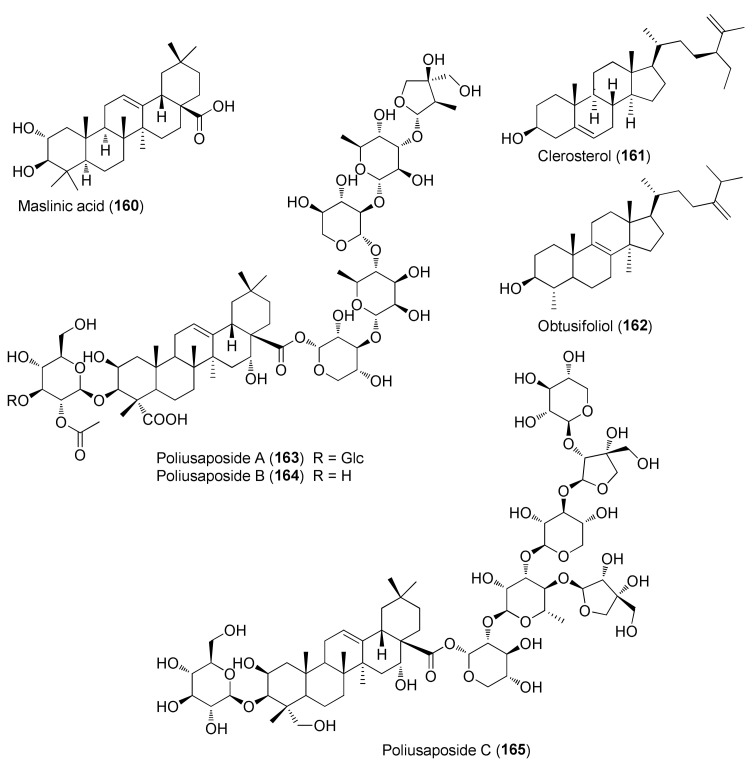
Representative triterpenoids and sterols isolated from *Teucrium polium*.

Seven taxa belonging to the genus *Teucrium*, native to the Kurdistan Region, Iraq (Table 1), are used for the preparation of remedies for various diseases in the local traditional medicine, as well as in other countries. In Figure 2, Figure 3, Figure 4, Figure 5, Figure 6, Figure 7, Figure 8, Figure 9, Figure 10 and Figure 11 and Table 1 and Table 2 of this first ethnopharmacological review dedicated to this group of Kurdish plants, we collected the structures of more than 190 representative specialized components of essential oils and extracts, their traditional uses and their various biological activities reported in the literature. In general, the components of most essential oils, mainly mono- and sesquiterpenoids, were determined, whereas non-volatile metabolites have been less investigated, except those from *T. polium* and *T. chamaedrys*. However, a great number of novel and bioactive furanoid *neo*-clerodane and *abeo*-abietane diterpenoids, sesquiterpenoids, triterpenoids, steroids, flavonoids, iridoids, phenylethanoids and other aromatic compounds were isolated. Thus, *Teucrium* taxa belonging to the Iraqi Kurdistan flora can be considered rich sources of compounds with the potential to develop efficacious therapeutic agents. This finding should stimulate further scientific investigations, as well as the implementation of measures to preserve the rich biodiversity of Kurdistan.

However, several studies have indicated the contemporaneous presence of substances with opposite biological effects, for example, antioxidant polyphenols and hepatotoxic *neo*-clerodanes. This finding should be seriously considered and an accurate screening for toxic substances should be compulsory, especially if the plants are used as raw materials for botanicals.

This example clearly indicates that better public and physician awareness through health education, early recognition and management of herbal toxicity and tighter regulation of complementary/alternative medicine systems are required to minimize the dangers of herbal product use [141]. Moreover, in vivo assays and evidence-based clinical trials are needed to confirm the therapeutic properties of bioactive compounds. Moreover, SAR and QSAR approaches on active metabolites should be implemented in further studies.

Based on the findings outlined in this review, we intend to further in-depth investigate the phytochemistry and biological activities of *Teucrium* species used in Iraqi Kurdistan, especially the poorly known species *T. melissoides*, *T. parviflorum* and *T. rigidum*. In this context, we consider it particularly important to exclude the presence of hepatotoxic *neo*-clerodane diterpenoids, considering the wide uses of *Teucrium* plants in the Kurdish traditional medicine.

## Figures and Tables

**Figure 12:**
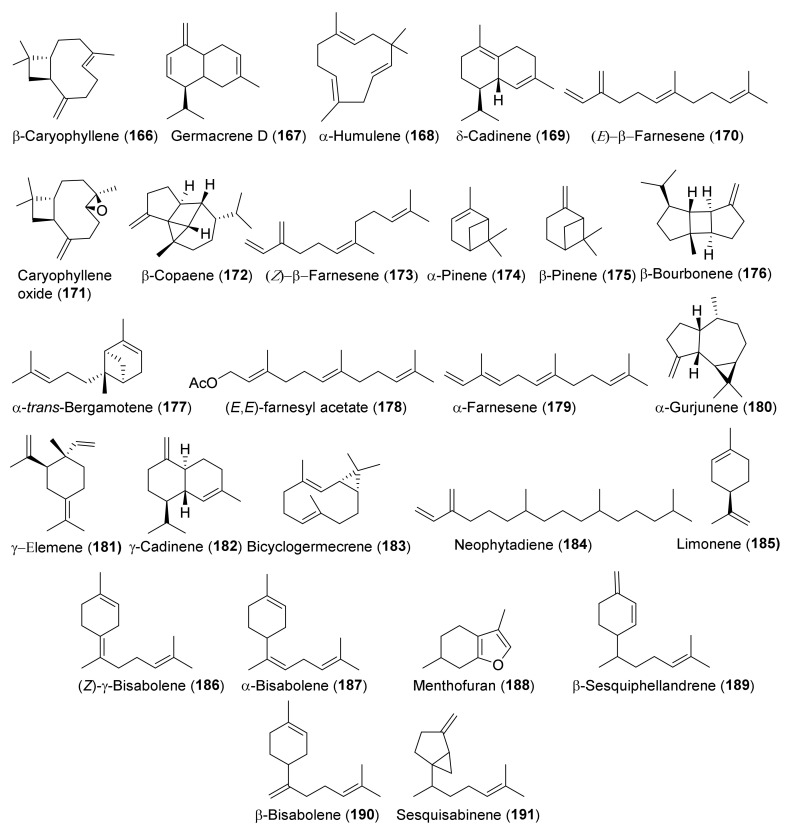
Representative terpenoids contained in essential oils isolated from *Teucrium* taxa.

**Table 1:** *Teucrium* taxa used in the traditional medicine of the Kurdistan Region, Iraq.

Botanical Name	Traditional Uses in the Kurdistan Region, Iraq	Growth Places (KRI Districts)
*T. chamaedrys* L.	Eaten as a digestive	Baradost, Mosul, Duhok, Gara, Sirsang, Sharanish, Zawita, Khantur, Atrush, Suwara Tuka, Ser Amadiyah, Qara Dagh
*T. melissoides* Boiss. & Hausskn. ex Boiss.	To treat abdominal diseases	Qandil, Pushtashan, Rowanduz, Avroman, Zawita
*T. oliverianum* Ging. ex Benth.	Antidiabetic remedy	Kirkuk, Hamrin
*T. parviflorum* Schreb.	To treat jaundice, liver disorders, stomachache and to reduce cholesterol level in blood	Amadiyah, Sulaimani, Rowandruz
*T. polium* L.	Antirheumatic and to treat abdominal pain	Arbil, Safin, Shaqlawa, Kirkuk, Jarmo, Mosul, Sharanish, Suwara Tuka, Zakho, Khantur, Gara, Atrush, Sirsang
*T. rigidum* Benth.	To treat abdominal problems	Darband-i Bazian
*Teucrium scordium* subsp. *scordioides* (Schreb.) Arcang. (synonym *T. scordioides* Schreb.)	Anti-inflammatory	Safin, Baradost, Zawita, Gara

**Table 2:** Traditional uses, biological activities and specialized metabolites isolated from *Teucrium* taxa used in the traditional medicines of the Kurdistan Region, Iraq, and other countries.

Taxon	Traditional Uses	Biological Activities	Secondary Metabolites
*Teucrium chamaedrys* L.	Astringent, antirheumatic, digestive, antispasmodic, anti-inflammatory, diuretic, diaphoretic, tonic, to treat wounds, fever, coughs, asthma, dyspepsia, anorexia, nasal catarrh, chronic bronchitis, gout, rheumatoid arthritis, abscesses, conjunctivitis, intermittent fever, uterine infections, to reduce body weight [29,31,32,33,34].	Hepatotoxicity [35], antimicrobial [36,37], antioxidant [37,38], phytotoxic [39] activities.	***Iridoids*** **1** [32] and **2** [32]. **Neo*-clerodane Diterpenoids*** **10** [32], **11** [40], **12** [41], **13** [40,41,42,43,44,45], **14** [39,46,47,48,49], **15** [46,48], **16** [47], **13** [31,40,44,45,49,50,51,52], **18** [31,45,52,53], **19** [31,39,41,43,45,47,48,51,52,54], **20** [31,39,45,46,47,48,52], teucrin C [51], teucrin D [51], **21** [46], **22** [39,47,49], **23** [31,39,45,47,49,52], **24** [39], **26** [43], **27** [43], **28** [40], **29** [31,39,52], **30** [31,39,45,46,47], **31** [31,45,52], **32** [43,55], **33** [55], **34** [39,56], **35** [47,57], **36** [47,57], **37** [57]. ***Flavonoids*** **87** [32], **88** [58], **89** [58], **90** [50], **91** [40], **98** [59], **99** [50]. ***Verbascoside Derivatives*** **107** [33,43,52,55,60,61], **108** [52] **109** [52], **111** [32], **112** [32], **113** [32], **114** [32,43,51]. ***Phenolic Compounds*** (*E*)-*p*-Coumaroyl-*O*-β-D-glucoside (**118**) [58]. ***Triterpenoids and Steroids*** 24α-Ethylcholesta-5,25-dien-3β-ol, β-sitosterol, α-amyrin, ursolic acid, 3β-hydroxystigmast-24(24^1^),25-dien-24^2^-al, 3β-hydroxy-24α-ethylcholesta-5,25-dien-7-one [50]. ***Essential Oil main components (%)* from aerial parts collected in Corsica (France)** **166** (29.0), **167** (19.4), **168** (6.8), **169** (5.4), **170** (4.4), **171** (3.2) [62]. **166** (33.9), **167** (18.5), **168** (7.5), **170** (5.1), **169** (4.6), **171** (3.1) [36]. ***Essential Oil main components (%)* from aerial parts collected in Croatia** **166** (47.6), **167** (29.0), **172** (5.7), **171** (4.5) [63]. ***Essential Oil main components (%)* from aerial parts collected in Iran** **167** (16.5), **173** (12.2), **166** (10.5), **174** (9.1), **169** (7.4), **175** (4.8), **176** (3.8), **177** (3.5) [64]. ***Essential Oil main components (%)* from aerial parts collected in Kossovo** **167** (24.1), hexadecanoic acid (12.1), **169** (7.0), linoleic acid (6.0), **166** (4.0), **178** (3.3), *n*-docosane (2.9) [65]. ***Essential Oil main components (%)* from aerial parts collected in Sardinia (Italy)** **166** (27.4), **167** (13.5), **171** (12.3), **168** (6.5), **174** (4.4), **175** (3.4), **176** (3.0) [62]. ***Essential Oil main components (%)* from aerial parts collected in Serbia-Montenegro** **166** (26.9), **167** (22.8), **168** (6.7), **171** (5.5), **174** (5.3), 3-octanol (3.7), **169** (3.1) [66]. ***Essential Oil main components (%)* from leaves collected in Turkey** **175** (13.1), **167** (9.5), **174** (8.9), **179** (8.0), **180** (7.8), **181** (7.4), **182** (6.4), heptacosane (4.8) [34]. ***Essential Oil main components (%)* from aerial parts collected in Turkey** **167** (32.1), **168** (14.2), **169** (13.1), **183** (6.7), **170** (4.3), **184** (4.1) [67].
*Teucrium melissoides* Boiss. & Hausskn. ex Boiss.	No traditional uses are reported in addition to the use indicated in Table 1.	No data have been reported	***Essential Oil main components (%*) from aerial parts collected in Iran** **174** (27.7), **175** (16.4), **185** (12.4), **167** (10.2), **166** (8.9), **186** (7.5), **168** (4.5) [68].
*Teucrium oliverianum* Ging. ex Benth.	Against diabetes [69,70,71,72].	Hypoglycemic [72]. Cytoprotective enzyme NAD(P)H: quinone oxidoreductase (NQO1) inducer activity [73].	***Iridoids*** **2** [72,73]. **Neo*-clerodane Diterpenoids*** **38** [72], **39** [72], **40** [72,73], **41** [72], **42** [71,72,73], **43**–**45** [72], **46** [74], **47** [70,72], **48** [70], **49** [70], **50** [74], **51** [71,72,73], **52** [71,72], **53** [74]. ***Flavonoids*** **87** [72], **92** [72,73]. ***Sterols*** 24(*S*)-Stigmasta-5,22,25-trien-3β-ol [72,73].
*Teucrium parviflorum* Schreb.	A decoction of the aerial parts is utilized in Turkey against hemorrhoids [75].	Antioxidant activity [76].	***Essential Oil main components (%*) from aerial parts collected in Turkey** **168** (18.6), **167** (9.2), **171** (8.8), **183** (6.0), **169** (4.5), **174** (4.4), **187** (4.4), **170** (3.7) [77].
*Teucrium polium* L.	Antiulcer, hypotensive, antispasmodic, anorexic and antipyretic agent, hypoglycemic, antidiabetic, hypolipidemic, anti-inflammatory, analgesic, antibacterial, antioxidant, digestive, antidiarrheal, anti-eczema, tonic, to treat skin diseases, stomach pain and disorders, gynecological diseases, kidney and liver diseases, hemorrhoids, menstruation disorders, toothache, body and joint pain, abortion, carminative, antimalarial, against rheumatism, cold and other diseases [26,78,79,80,81,82].	Anti-inflammatory [78,79], antispasmodic [79], hypotensive [69], hepatoprotective [79], acetylcholinesterase inhibitor and memory enhancer [79], antimutagenic [79], antioxidant and antiradical [79,82,83,84,85,86,87,88,89,90,91,92], antipyretic [93], antimicrobial [79,93,94,95,96,97], anticancer/cytotoxic [79,83,88,91,98,99,100], anti-ulcer [79,101], anti-nociceptive [79,102], antifeedant [103], hypoglycemic [79,89,104,105] hypolipidemic [79,106], proapoptotic [107], immunomodulatory [28], anticonvulsant [28] effects. Antioxidant [98] and references 210, 211, 226, and 255 in reference [26] of this review], antimicrobial [references 213, 214, 229, and 336] in reference [26] of this review].	***Iridoids and Iridoid Glycosides*** **2** [88,91], **3** [88,91,98,108], **4** [99], 1α-(β-D-glucopyranosyloxy)- 6α,7α-epoxy-4aβ,5α-dihydroxy-7-methyl-1,4a,5,6,7,7aβ-hexahydrocyclopenta[*c*]pyran (**5**) [99], 4-[(β-D-glucopyranosyloxy)methylene]-5α-(2-hydroxyethyl)-5-(α-L-rhamnopyranosyloxy)-3-methylcyclopent-2-en-1-one (**6**) [96], 4α-[(β-D-glucopyranosyloxy)methyl]-5α-(2-hydroxyethyl)-3-methylcyclopent-2-en-1-one (**7**) [96], 5α-[2-(β-D-glucopyranosyloxy)ethyl]-4α-hydroxymethyl-3-methylcyclopent-2-en-1-one (**8**) [96], 5α-(2-hydroxyethyl)-4α-hydroxymethyl-3-methylcyclopent-2-en-1-one (**9**) [96]. **Neo*-clerodane Diterpenoids*** **28** [91,100], **54** [80], **55** [80], **56** [85,100], **57** [100], **58** [91,100], **59** [104], teucrin P_2_ [109], **60** [103], **61** [103], **62** [103], **63** [109], **64** [110], **65** [100,109], **66** [91], **67** [91,100], **68** [91,100], **69** [109,111], **70** [100,109], **71** [109], **72** [108], **73** [110], **74**–**79** [91,100], **80** [100], **81** [112], **82** [112], **83** [85,91,100,113], **84** [114], **85** [114], **86** [100]. **Abeo*-abietane Diterpenes*** 12,16-Epoxy-6,11,14-trihydroxy-17(15→16)-*abeo*-3α,18-*cyclo*-5,8,11,13,15-abietapentaen-7-one (**129**) [90], 12,16-epoxy-6,11,14,17-tetrahydroxy-17(15→16)-*abeo*-3α,18-*cyclo*-5,8,11,13,15-abietapentaen-7-one (**130**) [90], 12,16-epoxy-6,11,14-trihydroxy-17(15→16)-*abeo*-5,8,11,13,15-abietapentaen-7-one (**131**) [90], 12,16-epoxy-6,11,14,17-tetrahydroxy-17(15→16)-*abeo*-5,8,11,13,15-abietapentaen-7-one (**132**) [90], 12,16-epoxy-6,11,14,15-tetrahydroxy-17(15→16)-*abeo*-3α,18-*cyclo*-5,8,11,13-abietatetraen-7-one (**133**) [90], 12,16-epoxy-6,11,14,15-tetrahydroxy-17(15→16)-*abeo*-5,8,11,13-abietatetraen-7-one (**134**) [91], **135–141** [90]. ***Sesquiterpenoids*** 7-*epi*-Eudesm-4(15)-ene-1β,6α-diol (**142**) [81], 7-*epi*-eudesrn-4(15)-ene-lβ,6β-diol (**143**) [81], **144** [81], **145** [81], (1*R*,4*S*,10*R*)-10,11-dimethyl-dicyclohex-5(6)-en-1,4-diol-7-one (**146**) [88], **147** [88], (10*R*,1*R*,4*S*,5*S*,6*R*,7*S*)-4,10-diepoxygermacran-6-ol (**148**) [88,95], 4β,5α-epoxy-7αH-germacr-10(14)-en-6β-ol-1-one (**149**) [95], 4β,5α-epoxy-7αH-germacr-10(14)-en,1β-hydroperoxyl,6β-ol (**150**) [95], 4β,5β-epoxy-7αH-germacr-10(14)-en,1β-hydroperoxyl,6β-ol (**151**) [95], 4α,5β-epoxy-7αH-germacr-10(14)-en,1β-hydroperoxyl,6α-ol (**152**) [95], **153** [95], eudesm-3-ene-1,6-diol (**154**) [95], *rel*-1β,3α,6β-trihydroxyeudesm-4-ene (**155**) [95], **156** [95], **157** [95], 4β,6β-dihydroxy-1α,5β(H)-guai-9-ene (**158**) [95], (1*R*,6*R*,7*R*,8*S*,11*R*)-1,6-dihydroxy-4,11-dimethylgermacra-4(5),10(14)-dien-8,12-olide (**159**) [88]. ***Flavonoids*** **87** [59,115,116,117], **90** [86,89,91,92,114], 3′,6-dimethoxy apigenin [86], 4′,7-*O*-dimethyl apigenin [86], **92** [116], **93** [85], **94** [95,115], **95** [59,91,92,114,116], **96** [114], **97** [114], **98** [58,96,114,116,118], **100** [86,89], **101** [85], **102** [85], **103** [85,92,118], **104** [59], 5,6-dihydroxy-7,4′-dimethoxyflavone [95], 5-hydroxy-7,4′-dimethoxyflavone [116], **105** [59], 3′,4′,5-trihydroxy-6,7-dimethoxyflavone [97], 5,6,7,3′,4′-pentahydroxyflavone [96], (**106**) [92], 5,7,4′-trihydroxyflavone 6,8-di-*C*-glucoside (vicenin-2) [118], apigenin 5-galloylglucoside [118]. ***Verbascoside Derivatives*** **110** [85], **114** [88,97,114], 2-(3-hydroxy-4-methoxyphenyl)-ethyl-*O*-(α-L-rhamnosyl)-(1→3)-*O*-(α-L-rhamnosyl)-(1→6)-4-*O*-(*E*)-feruloyl-β-D-glucopyranoside (**115**) [88], 2-(3,4-dihydroxyphenyl)-ethyl-*O*-(α-L-rhamnopyranosyl)-(1→3)-*O*-(α-L-rhamnopyranosyl)-(1→6)-4-[*O*-(*E*)-3-(4-hydroxy-3-methoxyphenyl)]-2-propenoate-β-D-glucopyranoside [88], 116 [85,88,91,95,97], **117** [97]. ***Phenolics, Lignans and Miscellaneous Compounds*** **119** [88], (*R*)-mandelonitrile-β-laminaribioside (**120**) [88], **121** [95], 2-(3,4-dihydroxyphenyl)ethanol (**122**) [96], 3,4-dihydroxy-3-(*O*-β-D-glucopyranosyl)phenylethanol (**123**) [96], 3-(*O*-β-D-glucopyranosyl)-α-(*O*-β-D-glucopyranosyl)-4-hydroxyphenylethanol (**124**) [96], (7*S*,8*R*)-4-(*O*-β-D-glucopyranosyl)-dehydrodiconiferyl alcohol (**125**) [96], (7*S*,8*R*)-5-methoxy-4-(*O*-β-D-glucopyranosyl)dehydrodiconiferyl alcohol (**126**) [96], **127** [114], **128** [114], α-tocopherol [119], β-arbutin [32]. ***Triterpenes, Sterols, Fatty Acids and Saponins*** Oleanolic acid [114], **160** [114], β-sitosterol [119,120], stigmasterol [119,120], campesterol [120], brassicasterol [120], **161** [119,120], **162** [119], linoleic acid [119], linolenic acid [119], palmitic acid [119], lauric acid [119], **163**–**165** [98]. ***Essential Oils*** The compositions of the essential oils isolated from aerial parts, leaves and flowers of *T. polium* collected in Algeria, Croatia, Francia, Greece, Iran, Jordan, Montenegro, Morocco, Oman, Russia, Saudi Arabia, Serbia, Syria, Tunisia and Turkey have been reported in several papers. See references [63,66,79,102,121,122,123,124,125,126,127,128,129,130] in this paper and references 210–259 in reference [26] of this review.
*Teucrium scordium* subsp. *scordioides* (Schreb.) Arcang.		Antimicrobial activity [131].	**Neo*-clerodane Diterpenoids*** **17** [131], **19** [131], **25** [131]. ***Essential Oil main components (%)* from aerial parts collected in Serbia** **188** (11.9), (*Z*)-octadec-9-enoic (oleic) acid (11.5), (*Z,Z*)-octadeca-9,12-dienoic (linoleic) acid (7.9), hexadecanoic (palmitic) acid (6.4), **166** (3.5), **127** (3.5) [132]. ***Essential Oil main components (%)* from aerial parts collected in Sicily (Italy)** **171** (25.8), **174** (19.4), **175** (8.5), 4-(1,5-dimethylhex-4-enyl)-cyclohex-2-enone (6.4), **189** (5.9), **166** (4.4), **190** (3.8), **191** (3.4) [133].

## Data Availability

Not applicable.
